# A specific flagellum beating mode for inducing fusion in mammalian fertilization and kinetics of sperm internalization

**DOI:** 10.1038/srep31886

**Published:** 2016-08-19

**Authors:** Benjamin Ravaux, Nabil Garroum, Eric Perez, Hervé Willaime, Christine Gourier

**Affiliations:** 1 Laboratoire de Physique Statistique, Ecole Normale Superieure/PSL Research University, UPMC Univ Paris 06, Université Paris Diderot, CNRS, 24 rue Lhomond, 75005 Paris, France; 2 Chimie ParisTech, PSL Research University, CNRS, Institut de Recherche de Chimie Paris (IRCP), F-75005 Paris, France

## Abstract

The salient phases of fertilization are gamete adhesion, membrane fusion, and internalization of the spermatozoon into the oocyte but the precise timeline and the molecular, membrane and cell mechanisms underlying these highly dynamical events are far from being established. The high motility of the spermatozoa and the unpredictable location of sperm/egg fusion dramatically hinder the use of real time imaging optical techniques that should directly provide the dynamics of cell events. Using an approach based on microfluidics technology, the sperm/egg interaction zone was imaged with the best front view, and the timeline of the fertilization events was established with an unparalleled temporal accuracy from the onset of gamete contact to full sperm DNA decondensation. It reveals that a key element of the adhesion phase to initiate fusion is the oscillatory motion of the sperm head on the oocyte plasma membrane generated by a specific flagellum-beating mode. It also shows that the incorporation of the spermatozoon head is a two steps process that includes simultaneous diving, tilt, and plasma membrane degradation of the sperm head into the oocyte and subsequent DNA decondensation.

Fertilization is the process by which a spermatozoon and an oocyte unite to produce a new individual, but many of its aspects are still poorly understood. To be able to fertilize the egg, sperm must attain a fertile state through the capacitation process and the subsequent acrosome reaction (AR). In mammals, this is done during the ascent to the oviduct ampulla, and the passage through the cumulus oophorus and the zona pellucida (ZP) surrounding the oocytes. However, they can also be induced in adequate medium *in vitro*. Interestingly, substantial progress in the understanding of the fertilization process came with the development of *in-vitro* fertilization assays using spermatozoa that were capacitated *in vitro* and oocytes from which cumulus oophorus and often ZP were removed before insemination. Phase contrast video-microscopy^1^,^2^ and electron microscopy images^3^ provided evidence for the salient phases of fertilization: (i) adhesion of the gametes, (ii) fusion of their membranes, (iii) internalization of the spermatozoon into the oocyte cytoplasm. The sperm regions involved in both the initial association of sperm and egg membranes and membrane fusion were determined^3^. An abundant literature discusses sperm motility as the indispensable motor that drives the sperm ascending in the female genital tract up to the locus of fusion on the oocyte membrane^4^,^5^,^6^,^7^. However, the flagellum movement was never really considered for its possible role in the subsequent gamete interaction phases i.e. adhesion and fusion. A sudden reduction of flagellum beating during gamete interaction is reported^1^ and this is considered as an indication that fusion process is underway. Insights in the molecular processes underlying fertilization events came with proteomic, genetics and biochemical approaches, even if the molecular and membrane mechanisms underlying membrane fusion are still unknown^8^,^9^. So far, four membrane proteins were proved to be essential: Izumo1 and Spaca6 on the sperm head^10^,^11^, Izumo1’s receptor Juno and Cd9 on the oocyte membrane^12^,^13^,^14^,^15^. At least three of them (Izumo1, Juno and Cd9) were shown to be involved in the prefusional gamete adhesion phase^12^,^16^,^17^,^18^, but the way they are involved in subsequent membrane fusion is still not elucidated. During the spermatozoon incorporation, some modifications of the oocyte membrane and of the underlying egg cortex were observed^19^,^20^, but no detailed overall picture of the gamete interaction region is established. Indeed, most of the data collected to elucidate these mechanisms correspond to snapshots while all the molecular mechanisms underlying the fertilization events are highly dynamical. Interestingly, state of the art optical techniques have the potential to directly provide the dynamics of cell events^21^,^22^. However such real time optical imaging techniques remain marginally used because of the high motility of the spermatozoa and the unpredictable location of sperm/egg fusion that hinder dramatically the use of these imaging techniques.

To overcome these problems, we developed an original experimental approach allowing to image in real time and with the best front view, the sperm/egg interaction zone from the onset contact of the gametes. Fertilization takes place in a microfluidic platform, in which a single spermatozoon is guided to a restricted and predefined region on the egg membrane where it can freely adhere and fuse in conditions as close as possible to physiological. The sperm/egg interaction zone can be accurately imaged in real time with state of the art optical microscopic techniques. With this approach, an accurate and reproducible timeline of the fertilization events was established. Surprisingly, it was observed that one specific mode of sperm flagellum beating is a necessary condition for gamete fusion. It was demonstrated that to undergo fusion, sperm must apply to the oocyte membrane an oscillating up and down stress by a high frequency oscillating movement of the sperm flagellum. After two minutes of these oscillations, the flagellum stops and one minute later, the membrane fusion occurs. The kinetics and features of the subsequent sperm internalization and nucleus decondensation were accurately obtained. The evolution of sperm membrane alteration during this process was visualized at micrometer scale. The time lag between sperm membrane degradation and DNA decondensation suggests a two steps process where the nuclear envelope disruption takes place in a late process independent from sperm membrane degradation. Our microfluidics approach guaranties the best frontal view of the sperm/egg interaction area, and combined with relevant fluorescent probe, it opens the way to the characterization of any membrane or cortex remodelling occurring in the area of interaction during fertilization.

## Results

To determine the timing of gamete interaction (adhesion, fusion and internalization) in mouse and to characterize the sequences of the different stages, we performed IVF assays in which one single acrosome reacted spermatozoon capacitated *in vitro* was selected and paired with depelucidated oocyte in metaphase II. Even if this experimental situation differs from the *in vivo* one by the absence of the oocyte ZP, it remains close to what happens *in vivo* once the fertilizing acrosome reacted sperm has crossed the ZP and starts to interact with the oolema of the metaphase II eggs. To be able to select acrosome reacted spermatozoa, we resorted to transgenic mice (Acr-EGFP) expressing Enhanced Green Fluorescent Protein in the sperm acrosome^23^,^24^. Indeed, during AR, acrosomal content among which EGFP is released. When illuminated with 488 nm wavelength that excites GFP, an acrosome intact spermatozoon emits green signal at the location of the acrosome of the spermatozoon head whereas an acrosome reacted one does not^23^,^24^. ZP free oocyte in metaphase II were selected on the basis of the presence of the first polar body. The oocytes were preincubated with Hoechst 33342, a dye excited at 405 nm, commonly used as an indicator that fusion has occurred^25^. Indeed, after membrane fusion, the dye present in the egg cytoplasm, can freely diffuse in the sperm head where it progressively binds to sperm DNA and generates increasing blue fluorescent signal.

### Specific oscillatory motion of the spermatozoon head during the adhesion phase is required for successful fertilization

**Figure 1:**
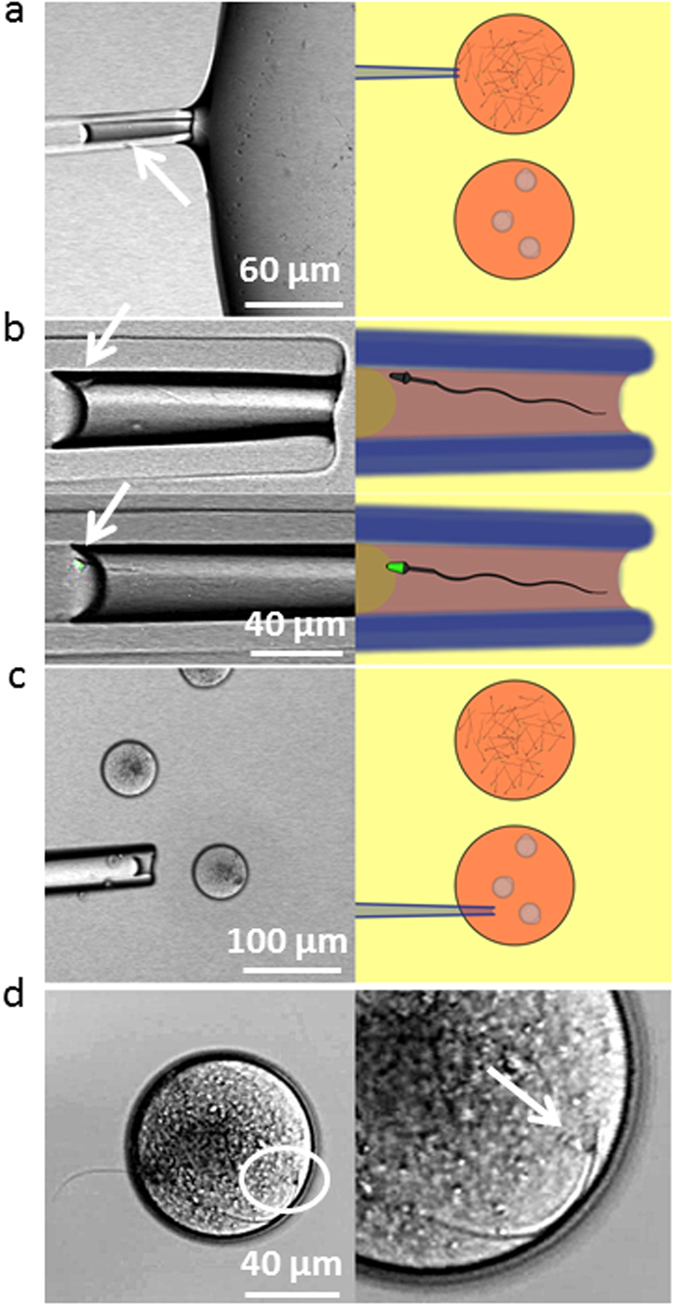
Principle of IVF assay with single sperm. The top M2 droplet contains capacitated Acr-EGFP sperm, the bottom droplet contains ZP-free oocytes in metaphase II preincubated with Hoechst 33342. (**a**)- A spermatozoon is trapped into a micropipette. (**b**)- Its acrosomal status is determined on the basis of EGFP fluorescence. (**c**)- An acrosome reacted spermatozoon (absence of EGFP signal) is released close to an oocyte. (**d**)- Interaction with the oocyte.

We performed individual IVF in a petri dish with two M2 medium droplets, one containing capacitated Acr-EGFP sperm and the other containing ZP-free eggs in metaphase II preincubated with Hoechst 33342 (Fig. 1a).

**Figure 2:**
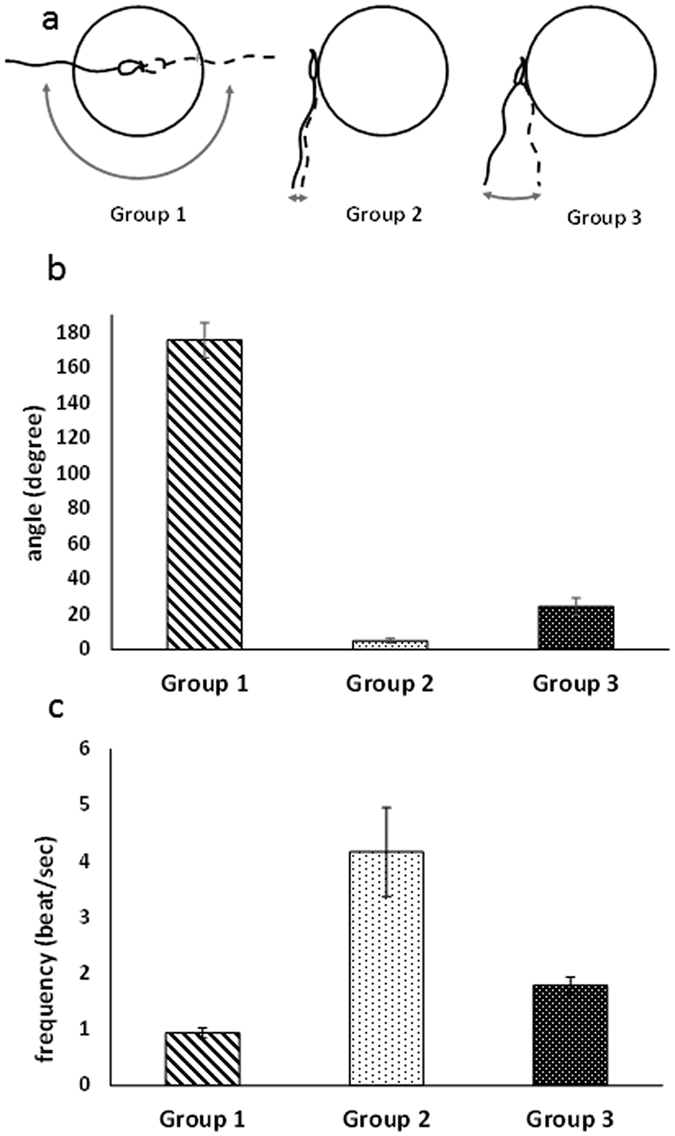
Flagellum beating modes of acrosome reacted spermatozoa interacting after sperm-egg contact. (**a**)- Group 1 strong whiplashed flagellum oscillations tangential to the oocyte, Group 2 low amplitude oscillations (group 2) (Video 2, Group 3 higher amplitude oscillations perpendicularly to the oocyte. (**b**)- angle spanned by the flagellum during beating oscillation, (**c**)- beating frequency.

**Figure 3:**
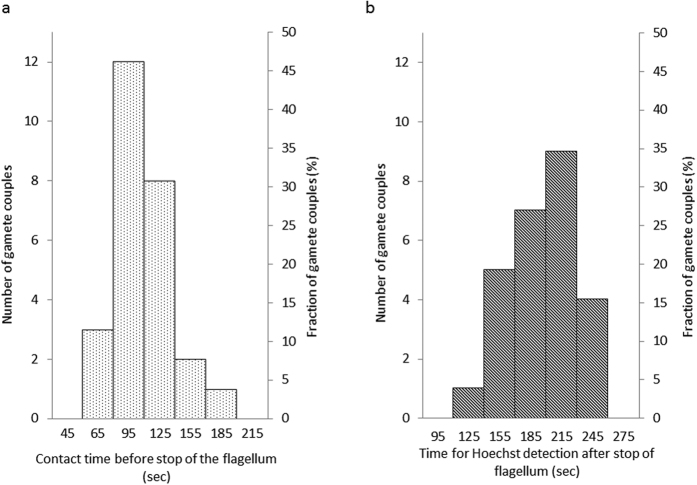
Stop of flagella beating and delays before visualization of Hoechst fluorescence for group 3 fertilizing spermatozoa. (**a**)- Distribution of time delays from onset of contact to end of flagellum beating. (**b**)- Distribution of time delays from end of the flagellum beating to detection of Hoechst spots on images.

The petri dish was placed on the stage of a confocal microscope equipped with a micromanipulator holding a micropipette. Based on the absence of EGFP fluorescence, a mobile spermatozoon was taken into the micropipette and transferred in the next droplet at the proximity of an oocyte with which it immediately started to interact (Fig. 1a–d). The fertilization status was determined on the basis of Hoechst observation on images. Even if all spermatozoa paired with an oocyte were acrosome reacted, only 29% of the paired gametes (26 gamete couples over 90) underwent fusion (Table 1). We however detected a variability of the flagellum movement of the spermatozoon that was correlated with the fusion rate. Indeed three distinct beating modes were observed (Fig. 2a): strong whiplashed flagellum oscillations tangential to the oocyte (group 1) (Video 1), low amplitude oscillations (group 2) (Video 2), and higher amplitude oscillations (group 3) (Video 3) perpendicularly to the oocyte. The angle spanned by the flagellum (175 ± 10 degrees for group 1, 5 ± 2 degrees for group 2 and 22 ± 6 degrees for group 3, p < 0,0001) and the beating frequency (0,9 ± 0,2 beat/sec for group 1, 3.2 ± 0,6 beats/sec for group 2 and 1,8 ± 0,2 beat/sec for group 3, p < 0,0001) are significantly different (Fig. 2b,c). While no sperm from group 1 (11 gamete couples) and from group 2 (28 gamete couples) succeeded to fuse, 52% of the spermatozoa from group 3 underwent fusion (26 gamete couples over 51) (Table 1). Interestingly, when the flagella of spermatozoa from group 3 had their movement hindered by hitting the middle of their flagella with a micropipette (22 gamete couples) or by being maintained in a micropipette (7 gamete couples), the fusion ability was inhibited (Table 1) even if the sperm head was laid on the oocyte membrane. These observations reveal a critical role of the of mode flagellum beating for fusion. Flagella oscillations imposed to the spermatozoon head different types of movements that were likely to influence gamete interaction. Indeed, for spermatozoa from group 1, the wide whiplash movement of the flagellum induced a rotation of the spermatozoon head, around a pivotal area located in its anterior part, tangentially to the oocyte membrane (Video 1 for side view, Video 5 for better resolved front view). The adhesion of the sperm head to the oocyte membrane through this tiny pivotal area was generally robust enough to resist to the strong driving force imposed by flagella whiplash beats, but not always. It happened occasionally that the spermatozoon detached from the oocyte membrane after a few minutes (2 times over 11 assays). Because of the rotating movement of the sperm head, its equatorial region at which the spermatozoon is described to fuse^3^,^26^, was mobile on the egg membrane. These conditions were therefore not favourable to the tight contact required for membrane fusion and could explain the systematic fusion failure of this group. For spermatozoa from group 2, the lazy undulations of the flagella did not seem to influence much the position of the sperm head that remained laid and immobile on the oocyte membrane. Its equatorial zone was in contact with the oocyte membrane, however fusion did not happen very rarely suggesting that sperm head motion on the oocyte membrane is a necessary condition for subsequent fusion. Spermatozoa from group 3, attached themselves to the oocyte membrane through the anterior part of their head after contact, like spermatozoa from group 1. However, in contrast to the non-fusional rotational movement of the head tangential to the membrane of group 1, the beating of the flagella imposed to the sperm head of group 3 to oscillate in a plane perpendicular to the oocyte membrane with its equatorial part alternatively pressed-on and pulled-off the oocyte membrane. During this oscillating period, it was often observed that the flagellum beating reduced significantly for a few seconds (~3 sec) before restoring its initial oscillation. For fertilizing couples, this oscillating phase never exceeded more than 180 sec. Indeed, after an average time of 113 ± 30 sec, the sperm flagellum suddenly and definitively stopped beating (Fig. 3a). Then, 202 ± 30 sec after this stop, fluorescent Hoechst spots, due to nuclear dyes transfer from the oocyte cytoplasm to the sperm DNA, were systematically observed in the spermatozoon head (Fig. 3a). These precursory indications that fertilization was in progress were confirmed by the penetration of the sperm head into the oocyte cytoplasm, the intensification and size increase of the Hoechst spot during the subsequent 80 minutes, the ejection of the second polar body 6 hours later, and the two cells division 24 hours later (Supplementary Figure S1).

### Guided fertilization with customized IVF microfluidic chip

**Figure 4:**
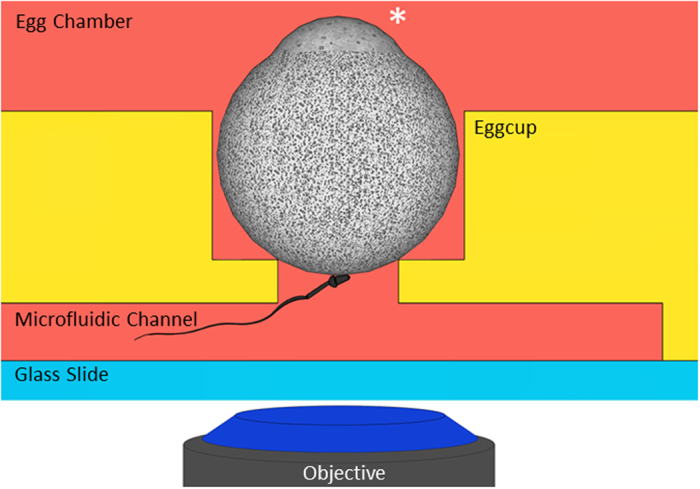
IVF in the microfluidic chip. The oocyte is maintained still in an eggcup equipped with a small opening at the bottom that communicates with a microfluidic channel. The star indicates the amicrovillar portion of the oocyte which is kept far from the opening on the channel. A selected acrosome reacted spermatozoon is introduced inside the channel and let free to interact with the restricted accessible membrane oocyte portion. The interaction zone is accurately imaged with a confocal microscope.

From the status of simple indicator of the fertilization, nuclear dye relocation has the potential to become a powerful tool to determine accurately the onset of gamete fusion and to characterize the dynamics of sperm internalization and DNA decondensation. However, this requires an accurate quantification of the temporal and spatial evolution of the fluorescent signal during the whole gamete interaction process. This is not an easy task because of the high motility of the spermatozoon and of the large size of the oocyte that make unpredictable the locus of interaction on the oocyte and that make the oocyte move erratically during the interaction process. To make such quantification possible, we have developed a microfluidic platform allowing to guide a spermatozoon to a predefined zone on the oocyte where the gamete interaction process can be imaged with optimal space and time resolution with confocal microscope. With this IVF microfluidic tool, an oocyte is maintained still in an eggcup equipped with a small opening at the bottom that communicates with a microfluidic channel (Fig. 4). When an acrosome reacted spermatozoon is introduced inside the channel, it reaches the oocyte through this small opening. The portion of membrane that is accessible to the spermatozoon (~300 μm^2^) is typically fifteen times larger than the sperm/egg interaction zone (~20 μm^2^). It is therefore large enough to welcome the fertilizing spermatozoon and yet represents only 1.5% of the total egg surface (20000 μm^2^). The oocyte is not able to escape from its tailored eggcup. The sperm is free to move and interact with the oocyte. These conditions therefore allow a very good front view of the sperm/egg contact area while imposing minimal constraints to the gametes.

IVF assays involving one single acrosome reacted spermatozoon and one single oocyte in metaphase II were conducted in this microfluidic chip. Spermatozoa with the three previously described types of flagella movements were tested (6 experimental days). In agreement with off-chip assays, all of them did adhere to the oocyte membrane. While 50% of the spermatozoa from group 3 (8 gamete couples) underwent fusion (Video 4), spermatozoa from group 1 (3 gamete couples) (Video 5) and from group 2 (4 gamete couples) did not fuse. For the fertilizing couples of group 3 (video 4), the characteristic times for beating to stop after contact (120 ± 30 seconds) and for the delay before Hoechst became detectable in the sperm head (215 ± 30 seconds after beating has stopped) were also fully consistent with those obtained off-chip. These results therefore confirmed the absence of bias of the microfluidic chip relative to off-chip experimental conditions and that the microfluidic chip does not impair gamete abilities. Last but not least, this customized IVF microfluidic chip guarantees a stable front view of the sperm/egg interaction zone. As the region of interest on the oocyte membrane is defined before the gamete encounter, all the setting parameters of the microscope can accurately be adjusted in advance. These valuable benefits make it possible to capture the sperm-egg interaction zone from the arrival of the sperm on the oocyte till its full internalization in conditions as close as possible to physiological with confocal imaging parameters allowing optimal front view of the fertilization process and an accurate quantification of the fluorescent signal.

From the onset of sperm/egg contact, series of 10 vertical confocal stacks with 1.5 μm increment were acquired continuously. A total thickness of 15 μm was imaged, starting from 1.5 μm under the bottom accessible membrane, up to a depth of 13.5 μm inside the egg cytoplasm in order to follow the interaction of the fertilizing spermatozoon with the oocyte membrane and its internalization and nucleus decondensation. The fluorescence signal (excitation 405 nm) was imaged and quantified stack-by-stack, and simultaneously bright field image sequences were recorded. Such a dual acquisition allowed observing the behaviour (movement) and morphological changes of the spermatozoon interacting with the oocyte while quantifying the time and space evolution of the Hoechst signal.

### Determination of the time of the onset of fusion

**Figure 5:**
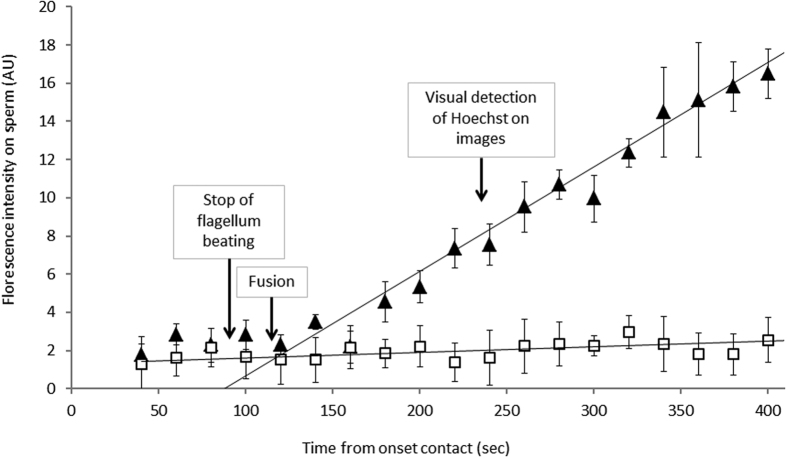
Determination of the fusion time. Typical time evolutions of the Hoechst transfer on the spermatozoon: for a couple of gamete that did not fertilize (squares), for a couple that did fertilize (triangles). The times for beating to stop, membrane fusion and Hoechst detection on images area are indicated with arrows.

Our first aim was to determine the onset of membrane fusion after contact. To do so, the fluorescent signal was quantified as a function of time in two regions of interest: one that includes the sperm/egg interface, and another one, close to it and of the same size, but that does not include the sperm/egg interface. The difference between both signals was the contribution of the spermatozoon to the fluorescence (Fig. 5). Two typical time evolutions of this signal were obtained depending whether the gamete underwent fusion (Fig. 5 triangles) or not (Fig. 5 squares). At the beginning of the gametes interaction, there is no significant difference between both signals. They show the same slow increase that may be attributed to membranes permeability to Hoechst dye that leads to leakages from the oocytes and thus to a weak spurious staining of the spermatozoon in contact with the oocyte. While this slow evolution continues for non-fertilizing couples (Fig. 5 squares), there is a sudden increase of the speed of Hoechst transfer to the sperm head for fertilizing couples (Fig. 5 triangles). This dramatic change of regime indicates that a fusion pore was formed, allowing the dye to diffuse from the oocyte cytoplasm to the spermatozoon head. The transition between the slow and fast regimes corresponds to time of membrane fusion. This fusion time was determined for each fertilizing couple. It systematically occurred within the minute that followed the stop of flagellum beating and around two minutes before the Hoechst signal was clearly identifiable on fluorescent images. These observations indicate that both the end of the beating and the observation of Hoechst are correlated to the gamete fusion but neither can be assimilated to the time of fusion itself.

### Internalization of spermatozoon, sperm membrane alteration and DNA decondensation kinetics

**Figure 6:**
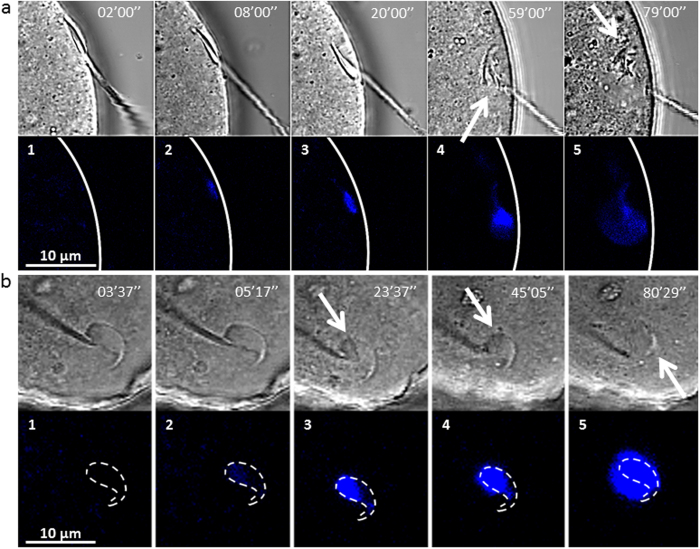
Snapshots of the sperm/egg interaction area during the fertilization process (bright field and fluorescence images, 405 nm wavelength). Timer indicates time after onset contact. Blue spots correspond to sperm DNA staining. (**a**)- Side view. a1-a3 Internalization and tilt of the spermatozoon head. a4-a5 sperm nucleus decondensation. Arrow on a4 indicates sperm membrane alteration. Arrow on a5 indicates the small portion of the spermatozoon head that remains intact after full decondensation. Flagellum seems to be stuck in the oocyte membrane. (**b**)- Front view with the microfluidic chip. The dashed line indicates the contour of the sperm head. b1-b3 Sperm DNA remains within these contours during 45 min. White arrow indicates sperm membrane alteration. b4-b5 the signal spreads out the contours which means that sperm DNA is decondensing. Arrow on b4 indicates sperm membrane alteration. Arrow on b5 indicates the small portion of the spermatozoon head that remains intact after full decondensation.

The next objective was to characterize the sequences of spermatozoon internalization during fertilization. Side view images obtained from off-chip experiments suggested that the penetration of the spermatozoon head subsequent to fusion was associated to a progressive tilt of the head from its initial tangential position on the membrane (Fig. 6a1 to a3) and that sperm DNA decondensation was coming as a second step after 50 min (Fig. 6a4 to a5). The evolution of the spermatozoon head during the first 100 min of fertilization was obtained with complementary front view and much better resolution with the microfluidic chip (Video 4, Fig. 5b1–b5).

**Figure 7:**
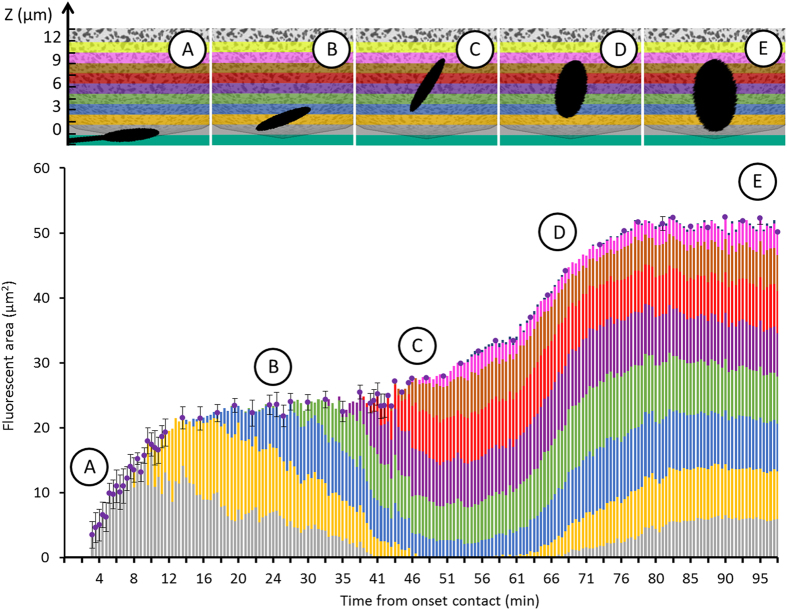
Evolution of the fluorescence 3D mapping allows to follow internalization and DNA decondensation kinetics. The dotted curve is the 2D projection area of the Hoechst spot in the horizontal plan. The coloured lines under the dot curve indicate in which layer(s) of the oocyte the fluorescence signal was detected. Each colour corresponds to one layer at one depth of the oocyte according to the scale given in the top banner. For each time, the full length of the line corresponds to 100% of the detected signal, and the colours segments that compose the line reflect the fraction of Hoechst per layer. The Top banner gives the representation of the internalization and decondensation phases reconstructed from the colour graph analysis.

The kinetics of sperm internalization and DNA decondensation were accurately determined by locating and quantifying the Hoechst signal within 10 distinct layers of 1.5 μm of the oocyte as a function time (Fig. 7). The dotted curve in Fig. 7 corresponds to 2D projection of the Hoechst spot in the horizontal plan and therefore provides the horizontal extension of stained DNA in μm^2^ during time. The coloured lines under the dotted curve indicate in which layer(s) of the oocyte the signal was detected. Each colour corresponds to a different layer of the oocyte. For each time, the full length of the line corresponds to 100% of the detected signal, and the colours segments that compose the line reflect the fraction of Hoechst per layer. The kinetics of sperm internalization and vertical extension of its DNA were therefore deduced from the time evolution of the colour waves.

The fluorescent signal due to sperm DNA staining started to be measurable 3 minutes after contact (Fig. 6b1 and dotted curve in Fig. 7A) in the layer near the oocyte membrane (grey colour Fig. 7A) meaning that the spermatozoon head was laying on the oocyte. Then, during 15 minutes the fluorescent spot increased in size (doted curve Fig. 7B) and in brightness (Fig. 6b2), reflecting the dynamics of Hoechst binding to the spermatozoon DNA. During this period, the fluorescent signal, first detected in this layer was shortly after also detected in the first and second layers (grey + yellow colours Fig. 7B) indicating the beginning of sperm internalization. From minutes 15 to 45, the fluorescent spot became brighter and brighter (Fig. 6b2–b4) meaning that dyes were still getting incorporated in the spermatozoon DNA but the spatial extension of the fluorescent spot stagnated around 22 μm^2^ as indicated by the plateau (dotted curve Fig. 7C). Since this surface typically corresponds to the surface of the longitudinal section of the spermatozoon head, these results suggested that during the 45 first minutes of contact, the spermatozoon DNA was maintained confined within the sperm head. During this time, the signal started being detected in the third layer (blue) then the fourth (purple) while progressively leaving the first and second layers (grey then yellow) (Fig. 7C). This succession of colours was the signature of the progressive sinking of the spermatozoon in the oocyte and the slight increase of the number of layers involving fluorescent signal at a given time confirmed the tilt of the head from its initial tangential position during its internalization (Fig. 7C), in agreement with off-chip side view (Fig. 6a1–a3). After 45 min of contact, the fluorescent spot started to spread. Within less than 30 min, its area doubled (dotted curve Fig. 7D). In the meantime, superficial layers that had previously lost some fluorescence because of sperm internalization, recovered it progressively, while symmetrically new deeper layers continued to acquire fluorescence (Fig. 7D). These behaviours were the signature that the DNA decondensation process was in progress. The end of the decondensation phase, 80 min after contact, was indicated by the stabilization of the fluorescent spot both in size and in position ((Fig. 7E).

The decondensation process did not seem to be directly correlated to the alteration of the spermatozoon membrane. Indeed, the first signs of membrane morphological changes observed in bright field appeared 15 min after contact with a clear dividing line at the frontier of the equatorial and post-equatorial segments (Fig. 6b3 arrow). This demarcation was followed by the progressive degradation of the post-equatorial membrane (holes in the membrane) (Fig. 6a4 arrows) and finally of the whole sperm membrane except for the top of the acrosome cap that remained easily visible in the egg even after decondensation completion (Fig. 6b5 arrow). When DNA spreading began 45 minutes after contact, membrane degradation was therefore already substantial, suggesting that the nuclear envelope disruption required for DNA spreading and the sperm plasma membrane degradation were independent processes.

## Discussion

This study demonstrates that not all acrosome reacted spermatozoa adhering to an oocyte can fuse and that a sperm specific movement during the gamete adhesion stage is essential in the establishment of gamete fusion conditions. If this movement (Group 3) is not produced, fusion is doomed to fail. The possibility that these spermatozoa showing such oscillatory movement have specific membrane features (like different molecular expression or membrane organization) that could intrinsically favour fusion was ruled out by control experiments showing that spermatozoa with suitable oscillating beats systematically failed to fuse when their movements were inhibited by introducing their flagellum into a micropipette or by hitting them (Table 1).

A gamete interaction zone submitted to the movement of the spermatozoon during an average time of 2 min therefore appears as a necessary condition for fusion in mouse. Such a requirement makes a major difference between the gametes and the cells involved in the other known cell-cell fusion processes in mammals (i.e. trophoblasts or myoblasts fusion)^27^,^28^,^29^,^30^. This difference may partly stem from the highly regionalized configuration of the spermatozoon membrane^31^. Indeed, by contrast to trophoblasts or myoblasts, a mere contact of the gametes anywhere on the spermatozoon head is not sufficient to ensure fusion. It has been known for a long time that the attachment of the spermatozoon to the oocyte membrane can take place on its anterior part but fusion cannot occur as long as the sperm equatorial domain does not come into intimate association with the oocyte surface^3^,^26^,^31^. Interestingly, we observed that the whiplash beating (video 1 and video 5), which leads to rapid changes of the location of the sperm equatorial zone, ruins the chances of establishing the intimate association required for membrane fusion. The reason why fusion is not observed in the cases of acrosome reacted spermatozoa with lazy movements (type 2 spermatozoa, Video 2) or convenient oscillating movements inhibited with micropipettes is however more questionable (Fig. 2a). Indeed, in both cases, the equatorial segment was apparently properly laying on the oocyte membrane. This suggests that membrane apposition of gametes is not sufficient to trigger lipid bilayers merging. The requirement of movement in this process suggests three non-exclusive pre-fusional membrane interaction mechanisms. The first one is that the up and down oscillating movement of the spermatozoon head perpendicularly to the oocyte membrane makes the sperm burrowing into the egg microvilli with the result to increase the chance to access hidden and potentially fusogenic receptors that would not be present on the microvilli extremities. Alternatively, the up and down oscillatory motion of the sperm head induces a sucking effect that progressively brings the membrane in the intimate contact required for fusion. Another possibility is that the oscillating mechanical constraints generated by the sperm beating on the gamete interface, induces a signal inside the oocyte that triggers membrane fusion.

Recently, several studies have attempted without success to induce cell-egg fusion by bringing in contact oocytes with somatic cells transfected with Izumo1 and/or Spaca6, the two only sperm proteins known to be essential for fertilization^16^,^17^,^32^. The idea was to specifically test the interaction of these proteins with the oocyte membrane but also to determine whether these proteins could be the minimum sperm molecular fusion machinery. The recurrent absence of fusion led to the conclusion that one or several other sperm factors were necessary for fusion^16^,^17^,^32^. In the light of our results, it can be speculated that one of the missing necessary factors for fusion is the driving oscillating force itself, resulting from flagella movement.

**Figure 8:**
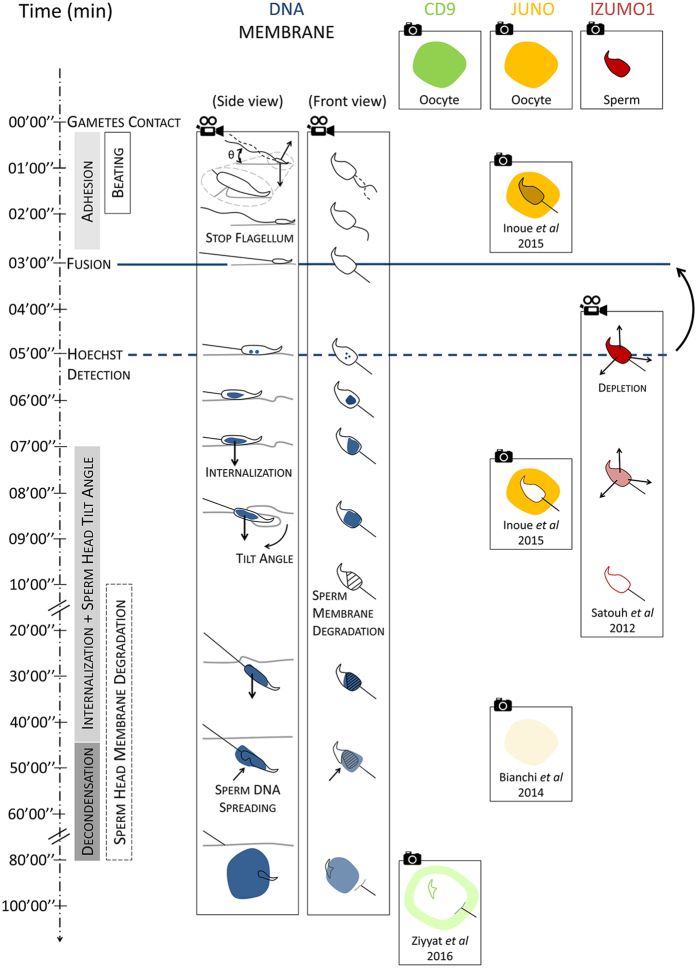
Timeline of fertilization events. The 3 first columns illustrate the results of this study. The first column summarizes the key events and their sequences during fertilization in mouse. The second and third columns report the evolution of the fertilizing spermatozoon from a side and front view, from onset contact with the oocyte till the full decondensation of its DNA regarding (i) its flagellum movement, (ii) its position relative to the oocyte membrane, (iii) its membrane degradation and (iv) Hoechst signal. Columns 4 to 6 give the state of the art knowledge regarding the localization of the essential proteins during fertilization (i) CD9^20^, (ii)Juno and (iii)^32^ Izumo1^21^,^32^. The blue line indicates the time of fusion onset, and the dashed blue line indicates the time at which Hoechst signal becomes clearly detected on images. The time lag between them illustrates the error made when Hoechst detection on images is assimilated to the onset of fusion. The video cameras indicate continuous and real time acquisitions while the cameras indicate that only snapshots are available.

Our microfluidic chip offers the possibility of controlling the location of gamete contact, to have it still during the whole interaction process and to simultaneously image in bright field and fluorescence the gamete interaction zone in real time. Thanks to this approach an accurate and continuous timeline of the fertilization process from the onset of gamete contact, to the full sperm DNA decondensation, was established for the first time in mouse (Fig. 8). The process begins with a first interaction phase during which the spermatozoon head oscillates up and down on the oocyte membrane because of flagellum beating. After 2 minutes in average, the flagellum stops beating once and for all. A sudden reduction of the flagellum movement is reported in several species and is considered as an indication that fusion is underway. However, our study shows that in mouse, membrane fusion comes soon after the beating has stopped (within the minute) but not simultaneously to it (Fig. 4). Indeed after the beating has stopped, the spermatozoon head, including its equatorial segment, remains in intimate apposition on the oocyte membrane. From the onset contact of gametes till their fusion, there is therefore a period of 3 min during which gametes interact to enable the fusion of their membranes. This key fusion event is followed by the internalization of the spermatozoon head. This phase lasts for approximatively 40 min, during which the spermatozoon head progressively dives into the oocyte while tilting from its initial position tangential to the oocyte membrane. The spermatozoon plasma membrane is getting more and more damaged but the spermatozoon DNA remains confined suggesting that its nuclear envelope is still intact. Indeed, it is 45 min after initial contact, while the post-equatorial part of the spermatozoon membrane damages are already advanced, that the decondensation process starts. Around 30 minutes are necessary to complete this phase. In the meantime, damage to the spermatozoon membrane still goes on, except for its anterior part that remains intact, consistent with a reminiscent sperm membrane vesicle already described^3^,^21^.

A main challenge of the mammalian fertilization current research is to elucidate the role of the four membrane proteins known to be essential in the fertilization process: Izumo1 and Spaca6 on the sperm head^10^,^11^,^12^,^13^,^14^,^15^, Juno and Cd9 on the oocyte membrane. Deep membrane reorganizations involving at least Izumo1, Juno and CD9 have been observed^12^,^20^,^21^,^32^. The sperm/egg interaction zone was shown to be enriched in Juno and Izumo1 before fusion and strongly depleted from this area after fusion (Fig. 7)^21^,^32^. Cd9 was shown to control the redistribution of some membrane proteins (Fig. 7)^20^. Nevertheless, the dynamics of these reorganizations are missing or incomplete, and the determination of the time of membrane fusion onset compared to the timing of these reorganizations was not accurately determined^21^,^32^. Moreover, in most of the experiments ZP-free oocytes were inseminated with a high quantity of sperm, leading to non-physiological situations with many spermatozoa interacting with the oocyte membrane. It is important to be able to distinguish the molecular and membrane events that occur before fusion from the ones occurring after fusion because the formers are likely to be the cause of membrane fusion while the latter result from it. Our microfluidic approach makes this distinction possible, since with well-targeted fluorescent probes, it can provide in the same experiment an accurate evaluation of the time of gamete fusion onset and a real time imaging of the membrane remodelling, from the onset of contact of a spermatozoon to the full sperm internalisation.

Beyond membrane reorganization, it was reported that the organization of oocyte actin is essential and required for sperm head internalisation and DNA decondensation^33^,^34^, but other ones showed no inhibitory effects of actin inhibitors^35^. The role of actin in the process of sperm entry in mammals is therefore not settled. Our approach could also be very helpful to the study of how the oocyte cytoskeleton evolves during fertilization to allow sperm DNA incorporation.

## Conclusion

Thanks to the possibility to perform controlled IVF assays involving one single spermatozoon, and to image with an optimal front view the sperm/egg interface from the first second of gamete encounter to the full sperm DNA decondensation, the present study refines and completes the global vision of the fertilization process that was acquired nearly 50 years ago. It reveals new insight on the essential role played by the sperm flagellum movement in the initial interaction phase that leads to fusion, and it brings an unparalleled temporal accuracy on the dynamics of the different phases of the spermatozoon internalization after fusion and DNA decondension. This microfluidic approach should pave the way to a systematic dynamical study of membrane and cortex remodelling and provide new insights on their role in the gamete fusion process and subsequent sperm incorporation.

## Methods

### Animals

All animal experimental protocols were approved by the Animal Care and Use Committee Charles Darwin, France (#3254), and Education and Research French Ministry (agreement number APAFIS#3254-2015121712165993 v2). The methods were carried out according to the European Community guideline (Directive 2010/63/EU) on the protection of animals used for scientific purposes.

### sperm

Sperm was obtained from 8–10 weeks old Acr - EGFP male mice (C57Bl6 background). It is a transgenic mouse line produced from mouse acrosin promoter which expresses EGFP in the sperm acrosome without diminishing fertilizing ability. The presence of EGFP in the acrosome facilitates observation of acrosomal integrity and to discriminate acrosome reacted spermatozoa from acrosome intact ones non-invasively^23^,^24^. Sperm were expelled from cauda epididymis and vas deferens into Ferticult® IVF medium (Fertipro, France) under mineral oil. Sperm were then incubated in Ferticult® at 37 °C, 5% CO_2_ in air for 2 hours to induce capacitation.

### oocytes

oocytes were obtained 6–8 weeks old wildtype female mice (C57Bl6 background, Charles River Laboratories, USA). Female mice were super-ovulated by intraperitoneal injections, first of 5 IU PMSG, followed by 5 IU hCG 48 hours apart. Cumulus-intact oocytes were collected into a Ferticult® medium drop 14 hours later by tearing the oviduct ampulla from sacrificed mice. Oocytes were separated from their cumulus by a brief incubation at 37 °C, in presence of hyaluronidase IV-S® (Sigma-Aldrich, USA) (15 mg.mL-1). Oocytes at metaphase II stage were selected on the basis of the presence of the first polar body. The Zona Pellucida (ZP) was subsequently removed by rapid treatment (<30 sec.) of the eggs with acidic Tyrode’s® (Sigma-Aldrich, USA) solution. Eggs were then incubated in Ferticult® for 2 hours at 37 °C, 5% CO_2_ in air to recover after the treatment. Oocytes were then incubated with Hoechst 33342 (Sigma-Aldrich, USA) at 1 μg.ml^−1^ for 5 minutes and washed.

### IVF microfluidic chip design

The IVF microfluidic chip is a PDMS 3D-platform sealed on a glass slide (Fig. 3). The top level is composed of a large open reservoir for eggs. In the bottom of the reservoir, there is a cylindrical recess where the oocyte to be fertilized is placed. This eggcup is tailored for the egg. Its diameter (80 μm) perfectly fits with the egg size so that the oocyte is trapped without be squeezed. Wedged in its egg-cup, the oocyte doesn’t move, even if an active spermatozoon adheres to it. This aspect is essential for reliable live imaging. The bottom of the eggcup is opened onto a thin cylindrical junction. The height of this junction is 10 μm, its diameter 30 μm. It makes the connection with the underneath channel in which the fertilizing spermatozoon is introduced. To maximize the chance of encounter, the channel has a dead end underneath the egg-cup that minimizes the probability for a spermatozoon to get lost far from the oocyte. The diameter of the junction is an essential aspect of the chip because it fixes the area of the egg membrane accessible to the sperm and therefore the surface to be imaged in order to make sure to capture the sperm/egg interaction site. This accessible surface corresponds to the bottom spherical cap of the oocyte (diameter 30 μm and height 3 μm).

### IVF assays of paired gametes

#### In a petri dish with a bottom glass slide

Two 10 μL M2 medium drops (M-7167, Sigma-Aldrich, USA) are covered with mineral oil to limit evaporation. 1 droplet contains capacitated Acr-EGFP sperm, the other contains ZP-free oocytes in metaphase II preincubated with Hoechst 33342. (Figure 1) The petri dish is placed on the stage of a confocal microscope, equipped with one micromanipulators holding micropipette (Narishige, Japan). One mobile acrosome reacted spermatozoon is selected on the basis of absence of EGFP fluorescence, trapped in the pipette and transferred in the second near an oocyte with which it is free to interact. The sperm/egg interaction was imaged in bright field and fluorescence during the whole fertilization process using an oil-immersion objective (HCX PL APO 40 × 1.25 Oil, Leica, Germany) and illuminated with 405 nm wavelength.

#### In the microfluidic chip

The microfluidic chip was filled with M2 medium and placed on the stage of a confocal microscope, equipped with two micromanipulators holding micropipettes (Narishige, Japan). (Figure 4) With one micropipette, a ZP-free oocyte, preincubated with Hoechst 33342, was positioned in the eggcup with its amicrovillar part at the opposite of the microfluidic channel (Fig. 3). This precaution was essential for mouse oocytes because their membranes are composed of two distinct parts: one rich in microvilli suitable for sperm/egg fusion and one smaller without microvilli, easily identifiable through its more convex shape, where fusion cannot happen^36^. With the second pipette, one acrosome reacted spermatozoon was introduced in the microfluidic channel and let free to interact with the accessible portion of the oocyte. The sperm/egg interaction was imaged in bright field and fluorescence during the whole fertilization process using an oil-immersion objective (HCX PL APO 40 × 1.25 Oil, Leica, Germany) and 405 nm illumination wavelength.

## Additional Information

**How to cite this article**: Ravaux, B. *et al*. A specific flagellum beating mode for inducing fusion in mammalian fertilization and kinetics of sperm internalization. *Sci. Rep.* **6**, 31886; doi: 10.1038/srep31886 (2016).

## Supplementary Material

Supplementary Video 1

Supplementary Video 2

Supplementary Video 3

Supplementary Video 4

Supplementary Video 5

Supplementary Figure S1

## Figures and Tables

**Table 1:** Fertilized status of paired gametes as a function of flagellum beating.

Experimental day	Group 1	Group 2	Group 3	Group 3 knocked	Group 3 immobilized
1	0 (3)	0 (6)	2 (4)	—	—
2	0 (2)	0 (3)	5 (9)	0 (6)	—
3	—	0 (5)	4 (9)	0 (3)	—
4	0 (3)	—	7 (9)	0 (7)	—
5	0 (1)	0 (4)	3 (8)	0 (6)	—
6	0 (2)	0 (4)	1 (6)	—	—
7	—	0 (5)	2 (3)	—	—
8	—	0 (1)	2 (3)	—	—
9	—	—	—	—	0 (3)
10	—	—	—	—	0 (4)
Total	0 (11)	0 (28)	26 (51)	0 (22)	0 (7)

Each experimental day, the sperm and the oocytes were collected from one male and one or two super ovulated females. Oocytes were paired with one single acrosome reacted spermatozoon and each gamete couple was sorted in 3 groups as a function of sperm flagellum movement when interacting with the oolema. Group 1: strong whiplashed flagellum oscillations tangential to the oocyte, Group 2 low amplitude oscillations, Group 3 higher amplitude oscillations perpendicular to the oocyte. Group 3 Knocked corresponds to Group 3 with flagellum knocked to stop its beating, Group 3 Immobilized corresponds to Group 3 with flagellum movement hindered by being confined in a micropipette. The first number without parenthesis corresponds to the number of fused couples, the number between parentheses is the number of couples.
