# Supplementary material for: A specific flagellum beating mode for inducing fusion in mammalian fertilization and kinetics of sperm internalization
Source: Sci Rep. 2016 Aug 19;6:31886. doi: 10.1038/srep31886 (PMC4990900; doi:10.1038/srep31886)
Supplement: Supplementary Figure S1 [file srep31886-s6.pdf]

Supplementary information

A specific flagellum beating mode for inducing fusion in mammalian fertilization and kinetics of sperm internalization

Benjamin Ravaux[1], Nabil Garroum[1], Eric Perez[1], Hervé Willaime[2], Christine Gourier[1]

1 Laboratoire de Physique Statistique, Ecole Normale Superieure/ PSL Research University, UPMC Univ Paris 06, Université Paris Diderot, CNRS, 24 rue Lhomond, 75005 Paris, France

2 Chimie ParisTech, PSL Research University, CNRS, Institut de Recherche de Chimie Paris (IRCP), F-75005 Paris, France

Corresponding author: gourier@lps.ens.fr

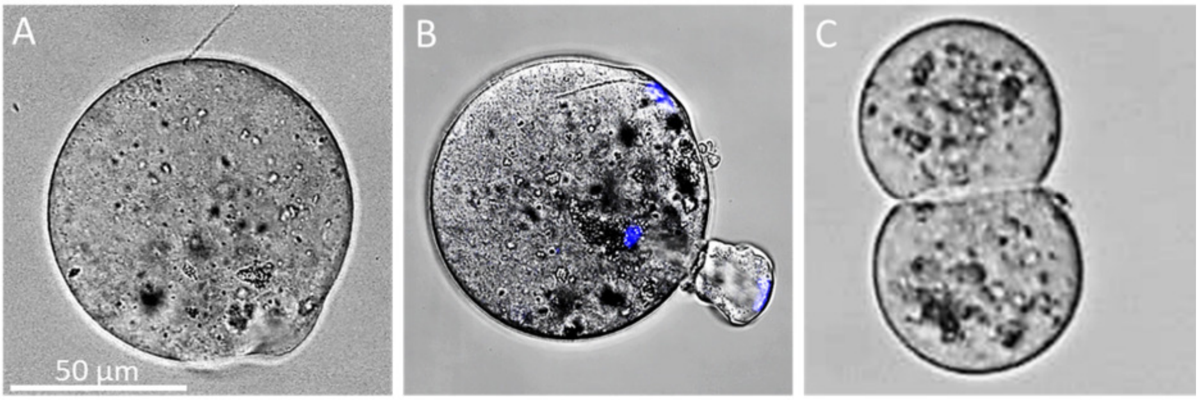

Figure S1. Evolution of In Vitro Fertilizations between one spermatozoon and one oocyte. A) After 2 hours. Membrane remodelling has occurred at the bottom and flagellum is pinned on the top. B) After 6 hours. The egg emits its second polar body. The decondensed sperm DNA is detected at the top of the oocyte by the poesence of the Hoechst dye. A fragment of the flagellum is still there. C) After 24 hours. 2 cells division.

Video legends

Video 1 : Strong whiplashed flagellum oscillations tangentially to the oocyte membrane (group 1) of an acrosome reacted spermatozoon interacting with an oocyte. Can not fertilize

Video 2 low amplitude oscillations (group 2) perpendicularly to the oocyte membrane of an acrosome reacted spermatozoon interacting with an oocyte. Can not fertilize.

Video 3 vigorous oscillation (group 3) perpendicularly to the oocyte membrane of an acrosome reacted spermatozoon interacting with an oocyte. Fertilize.

Video 4 Sequence of sperm internalization and DNA decondensation. Left bright field time lapse images. Right time lapse confocal images. Timer indicates time after onset of sperm/egg contact.

Video 5 Front view in the microfluidic chip of whiplashed flagellum oscillations tangentially to the oocyte membrane (group 1) of an acrosome reacted spermatozoon interacting with an oocyte. Can not fertilize.
